# Serum ferritin levels are associated with advanced liver fibrosis in treatment-naive autoimmune hepatitis

**DOI:** 10.1186/s12876-022-02098-z

**Published:** 2022-01-17

**Authors:** Qingling Chen, Min Gao, Hang Yang, Ling Mei, Rui Zhong, Ping Han, Peiyan Liu, Lili Zhao, Jing Wang, Jia Li

**Affiliations:** 1 Department of Hepatology, Second People’s Clinical College of Tianjin Medical University, Tianjin, China; 2 Department of Hepatology, Tianjin Second People’s Hospital, No. 7, Sudi South Road, Nankai District, Tianjin, 300192 China; 3 Department of Neurology, The First Hospital of Jilin University, Changchun, Jilin China

**Keywords:** Autoimmune hepatitis, Ferritin, Iron metabolism disorders, Liver fibrosis

## Abstract

**Background and aim:**

The association between iron-metabolism-related variables and liver fibrosis in chronic hepatitis C and nonalcoholic fatty liver disease is now well known. However, the relationship has not been extensively studied in autoimmune hepatitis (AIH). We aimed to investigate the association between variables associated with iron metabolism and advanced liver fibrosis among untreated patients with AIH.

**Methods:**

Ninety-seven untreated AIH patients were enrolled in this cross-sectional study. All participants underwent iron metabolism index detection and liver biopsy. Multiple logistic regression analysis was used to explore the association of iron-metabolism-related variables with advanced liver fibrosis.

**Results:**

Among the 97 AIH patients, 38 (39.2%) had advanced liver fibrosis, and 59 (60.8%) did not. In multivariate logistic regression analysis, immunoglobulin G (odds ratio [OR], 1.123; 95% confidence interval [CI] 1.023–1.232, *P* = 0.014), platelet count (OR 0.988; 95% CI 0.979–0.997, *P* = 0.013), prothrombin time (OR 1.758; 95% CI 1.143–2.704, *P* = 0.010) and ferritin (OR 1.002; 95% CI 1.001–1.004, *P* = 0.012) were independent risk factors for predicting advanced liver fibrosis in AIH patients.

**Conclusion:**

Higher serum ferritin was independently associated with advanced liver fibrosis among patients with treatment-naive AIH.

## Introduction

Autoimmune hepatitis (AIH) is a chronic and progressive inflammatory liver disease characterized by elevated levels of serum aminotransferases and immunoglobulin G (IgG), the presence of autoantibodies, and interface hepatitis with lymphoplasmacytic infiltration in liver histology [1]. AIH is a heterogeneous condition that occurs globally in children and adults of all ages and affects all ethnicities, with a female predominance. The prevalence of AIH is significantly different among different geographical regions and ethnic populations, and it is higher in North America and Europe than in the Asia–Pacific area [2–4]. Although previous AIH studies were mostly conducted in Europe and North America, an increasing number of studies from eastern countries have been reported in the past 10 years [3]. Due to the lack of specific diagnostic markers in AIH patients, most patients have significant fibrosis or even cirrhosis at the initial presentation [5]. An evaluation of the liver fibrosis stages is essential for the selection of treatment strategies and the estimation of the long-term prognosis for AIH patients.

Liver biopsy is considered the gold standard for the assessment of liver fibrosis in AIH patients [6, 7]. However, the clinical application of liver biopsy is often limited by its invasiveness, high cost, sampling errors, risk of complications, and poor patient compliance, particularly in the follow-up period [8, 9]. Therefore, it is important to develop noninvasive and convenient markers capable of accurately evaluating the stages of fibrosis in AIH patients. Indeed, several noninvasive markers for assessing liver fibrosis and cirrhosis have been applied in clinical practice, including the fibrosis index based on 4 factors (FIB-4), the aspartate transaminase-to-platelet ratio index (APRI), the aspartate aminotransferase/alanine aminotransferase ratio (AAR), and the AAR/platelet ratio index (AARPRI) [10, 11]. These noninvasive markers can be used to detect cirrhosis in patients with chronic viral hepatitis and nonalcoholic fatty liver disease. However, the ability of these markers to detect early liver fibrosis and cirrhosis in AIH patients remains uncertain. In recent years, liver stiffness measurement (LSM) has been widely investigated as a noninvasive method to quantify liver fibrosis, especially in metabolic and viral liver diseases [12, 13]. Nevertheless, the application of LSM has not been widely investigated in patients with AIH. Hartl et al. [14] demonstrated that transient elastography is a reliable tool for the detection of liver fibrosis in treated patients with AIH, but it showed limited value in therapy-naive individuals with active hepatitis.

Disturbances in iron regulation are reported in diverse chronic liver diseases beyond haemochromatosis and are mostly associated with a worse disease course [15–22]. Hyperferritinaemia has been the main manifestation of disturbed iron homeostasis in chronic liver diseases [16–19]. Elevated serum ferritin levels were reported to be independently associated with advanced liver fibrosis in patients with chronic hepatitis C and nonalcoholic fatty liver disease [23–27]. However, to date, the relationship between iron dysregulation and liver fibrosis has not been extensively studied in patients with AIH. Therefore, we analysed the status of the iron-metabolism-related variables and compared them with fibrosis stages in liver biopsy specimens in a cohort of untreated AIH patients. Nevertheless, there is usually increased inflammation in the liver with acute phase reaction at diagnosis, and ferritin levels can reflect inflammation; therefore, we will pay more attention to these same variables in treated patients after remission, which could valuable for estimations of disease progression, in future research.

## Methods

### Study population

In this retrospective study, we reviewed the medical records of adult AIH patients who underwent liver biopsy prior to immunosuppressive therapy between March 2013 and March 2021 in the Department of Hepatology, Tianjin Second People’s Hospital, Tianjin, China. The AIH diagnosis was made if patients met the criteria of (1) a 1999 revised International Autoimmune Hepatitis Group (IAIHG) score ≥ 10 and/or (2) an 2008 IAIHG simplified AIH score ≥ 6 [28, 29]. Patients with primary biliary cholangitis, primary sclerosing cholangitis, autoimmune overlap syndromes, viral hepatitis, alcoholic or nonalcoholic fatty liver disease, drug-induced liver disease, hereditary metabolic liver disease, hereditary haemochromatosis, hepatocellular carcinoma, and severe systemic diseases were excluded. Finally, a total of 97 AIH patients were included in this cross-sectional analysis.

This study protocol was approved by the Ethics Committee of Tianjin Second People’s Hospital and conducted according to the principles of the Declaration of Helsinki. We obtained written informed consent from all patients.

### General characteristics and laboratory test results

The medical records of all enrolled patients diagnosed with AIH were reviewed. Demographic data (age and sex) and laboratory results (of routine blood tests, biochemistry tests, and immunology tests) were collected and analysed. For all patients, blood samples were collected on the same day within 1 week before liver biopsy. Serum alanine aminotransferase (ALT), aspartate aminotransferase (AST), alkaline phosphatase (ALP), gamma-glutamyl transpeptidase (GGT), total bilirubin (TBIL), albumin (ALB), and IgG were detected by a Hitachi 7180 Automatic Biochemical Analyser (Hitachi, Ltd, Tokyo, Japan). The cut-off values for each parameter were set as values above the upper limit of the normal range, i.e., at 50 U/L for ALT, 40 U/L for AST, 125 U/L for ALP, 60 U/L for GGT, 20.52 μmol/L for TBIL, 55 g/L for ALB, and 16 g/L for IgG. Antibodies were assayed by indirect immunofluorescence or immunoblotting using a CycleBlot 48 automatic Western blotting instrument, and the starting dilution was 1:100. The complete blood count was measured using a Sysmex XN-2000 haematology analyser (Sysmex corporation, Kobe, Japan) according to the manufacturer’s recommendation. The coagulation tests were performed by the clotting method on the automatic coagulometer "STAGO Compact" ("Diagnostica Stago", France).

All the patients were assayed for variables related to iron metabolism: serum iron (colorimetric test), transferrin (immunoturbidimetry), ferritin (latex-enhanced immunoturbidimetric method), and unsaturated iron binding capacity (UIBC) (colorimetric test) following standard laboratory procedures. Subsequently, total iron binding capacity (TIBC) was calculated as UIBC + iron, and the transferrin saturation index (TSI) was calculated as serum iron/TIBC × 100.

### Histological assessments

Ultrasound-guided liver biopsy was performed using a 16-gauge disposable needle for all AIH patients under local anaesthesia. The liver pathology diagnosis required a liver specimen of at least 1.0 cm, which contains a minimum of 10 portal tracts. Each obtained specimen was fixed in 10% neutral-buffered formalin, embedded in paraffin, and routinely stained with haematoxylin–eosin and Masson’s trichrome. Liver tissue specimens were independently evaluated by two experienced liver pathologists blinded to patient clinical characteristics. Typical features of AIH include interface hepatitis, lymphocytic/lymphoplasmocytic infiltration in portal tracts, and hepatic rosette formation [29]. The histological assessment of the inflammatory grades and the fibrosis stages were based on the Batts–Ludwig scoring system [30, 31]. Liver fibrosis was classified into the following 5 stages: F0, no fibrosis; F1, portal fibrosis without septa; F2, portal fibrosis with few septa; F3, numerous septa without cirrhosis; and F4, cirrhosis [31]. Stages F3–F4 are defined as advanced liver fibrosis.

### Statistical analysis

We tested whether the continuous variables analysed showed a normal distribution using the Kolmogorov–Smirnov test. Normally distributed values are expressed as the mean (± standard deviation), while nonnormally distributed values are expressed as the median (quartile 25, quartile 75). Data for categorical variables are presented as the number (n) and proportion (%). Data were analysed by Spearman’s coefficient of correlation (r), Student’s t test, the Mann–Whitney U test and the chi-square test where applicable. The association between variables and advanced liver fibrosis was assessed by binary logistic regression analysis. Univariate logistic regression analysis was performed by the “Enter” method, and the variables that were statistically significant were entered into multivariate logistic regression analysis. The receiver operating characteristic (ROC) curve of ferritin was analysed to determine the best cut-off value for the prediction of advanced fibrosis stage by the DeLong method. Statistical analyses were performed using SPSS Statistics 26 (IBM, New York, NY, USA) and MedCalc Statistical Software version 15.8 (MedCalc Software bvba, Ostend, Belgium; https://www.medcalc.org; 2015). A two‐sided *P* < 0.05 was considered statistically significant.

## Results

### General characteristics of the patients

A total of 97 AIH patients with a mean age of 54.0 years were included in this study. The majority of patients were female (84.5%), and the prevalence of autoantibodies was 85.6%. The median levels of IgG, ALT, and AST were 16.80 (13.35, 21.10) g/L, 88.0 (32.0, 322.5) U/L, and 113.0 (35.0, 401.0) U/L, respectively. The distribution of patients according to liver fibrosis stage is as follows: F0, 11 (11.3%) patients; F1, 22 (22.7%) patients; F2, 26 (26.8%) patients; F3, 21 (21.6%) patients; and F4, 17 (17.5%) patients. Further information on the characteristics of the patients is shown in Table 1.

**Table 1:** General characteristics of the patients with autoimmune hepatitis

Variables	n = 97
Age (years)	54.0 ± 11.3
Female, n (%)	82 (84.5)
Autoantibodies positive, n (%)	83 (85.6)
ANA + , n (%)	78 (80.4)
ASMA + , n (%)	24 (24.7)
Anti-LKM1 + , n (%)	3 (3.1)
Anti-LC1, n (%)	2 (2.1)
Anti-SLA/LP, n (%)	2 (2.1)
IgG (g/L)	16.80 (13.35, 21.10)
ALT (U/L)	88.0 (32.0, 322.5)
AST (U/L)	113.0 (35.0, 401.0)
ALP (U/L)	110.0 (75.5, 151.0)
GGT (U/L)	125.9 (59.0, 248.0)
TBIL (μmol/L)	20.0 (12.7, 68.4)
ALB (g/L)	40.1 ± 5.6
PLT (× 10^9^/L)	184.8 ± 70.5
PT (s)	13.2 ± 1.5
Iron (μmol/L)	27.7 ± 14.4
Transferrin (g/L)	2.67 (2.28, 3.17)
Ferritin (μg/L)	214.0 (78.0, 515.0)
UIBC (μmol/L)	31.2 (21.2, 48.2)
TIBC (μmol/L)	59.8 (53.8, 68.9)
TSI (%)	43.6 (28.2, 64.2)
AIH score (1999)	15.8 ± 2.7
AIH score (2008)	7.5 ± 1.3
Histology (inflammation), n (%)	
G0	0 (0)
G1	3 (3.1)
G2	42 (43.3)
G3	40 (41.2)
G4	12 (12.4)
Histology (fibrosis), n (%)	
F0	11 (11.3)
F1	22 (22.7)
F2	26 (26.8)
F3	21 (21.6)
F4	17 (17.5)

Data are expressed as mean (± standard deviation), median (quartile 25, quartile 75) or number (proportion)

*ANA* antinuclear antibody, *ASMA* anti-smooth muscle antibody, *Anti-LKM1* anti-liver kidney microsomal antibody type 1, *Anti-LC1* anti-liver cytosol type 1 antibody, *Anti-SLA/LP* anti-soluble liver antigen or anti-liver-pancreas antibodies, *IgG* immunoglobulin G, *ALT* alanine aminotransferase, *AST* aspartate aminotransferase, *ALP* alkaline phosphatase, *GGT* gamma-glutamyl transpeptidase, *TBIL* total bilirubin, *ALB* albumin, *PLT* platelet, *PT* prothrombin time, *UIBC* unsaturated iron binding capacity, *TIBC* Total iron binding capacity, *TSI* transferrin saturation index

### General characteristics of patients by liver fibrosis stage

The included patients were divided into an advanced fibrosis group and a nonadvanced fibrosis group according to the results of liver biopsy analysis. Among the 97 AIH patients, 38 (39.2%) had advanced liver fibrosis, and 59 (60.8%) did not. No significant difference in sex proportion was observed between the two groups (*P* = 0.943), but the age of patients in the advanced fibrosis group was significantly higher than that in the nonadvanced fibrosis group (*P* = 0.010). The IgG, TBIL, prothrombin time (PT), ferritin, and TSI in the advanced fibrosis group were significantly higher than those in the nonadvanced fibrosis group (*P* < 0.05), while the ALB, platelet (PLT) count, transferrin, UIBC, and TIBC in the advanced fibrosis group were significantly lower than those in the nonadvanced fibrosis group (*P* < 0.05). Significant differences between the two groups were not observed for other parameters (*P* > 0.05), as shown in Table 2.

**Table 2:** General characteristics of subjects by liver fibrosis stage

Variables	F0–2 (n = 59)	F3–4 (n = 38)	*P*
Age (years)	51.7 ± 11.7	57.5 ± 9.7	0.010
Female, n (%)	50 (84.7)	32 (84.2)	0.943
Autoantibodies positive, n (%)	48 (81.4)	35 (92.1)	0.141
IgG (g/L)	15.00 (12.90, 19.18)	18.70 (15.85, 24.60)	0.002
ALT (U/L)	79.0 (29.0, 387.0)	122.5 (39.8, 311.3)	0.414
AST (U/L)	69.0 (30.0, 387.0)	173.0 (45.8, 421.0)	0.093
ALP (U/L)	101.0 (71.0, 151.0)	118.0 (78.5, 161.0)	0.206
GGT (U/L)	122.0 (73.0, 184.0)	132.0 (54.3, 298.5)	0.444
TBIL (μmol/L)	17.4 (11.8, 33.5)	28.0 (14.8, 134.6)	0.026
ALB (g/L)	41.1 ± 5.0	38.6 ± 6.2	0.035
PLT (× 10^9^/L)	208.5 ± 68.2	148.1 ± 57.5	< 0.001
PT (s)	12.7 ± 1.2	13.9 ± 1.6	< 0.001
Iron (μmol/L)	25.9 ± 16.0	30.5 ± 11.0	0.095
Transferrin (g/L)	2.75 (2.32, 3.25)	2.42 (2.13, 2.88)	0.022
Ferritin (μg/L)	124.0 (43.0, 306.0)	354.0 (189.5, 865.5)	< 0.001
UIBC (μmol/L)	39.3 (23.3, 51.6)	25.0 (16.2, 32.9)	0.005
TIBC (μmol/L)	64.4 (56.1, 72.4)	55.9 (51.7, 61.5)	0.004
TSI (%)	33.8 (22.9, 58.8)	55.1 (42.2, 69.4)	0.005

Data are expressed as mean (± standard deviation), median (quartile 25, quartile 75) or number (proportion)

*IgG* immunoglobulin G, *ALT* alanine aminotransferase, *AST* aspartate aminotransferase, *ALP* alkaline phosphatase, *GGT* gamma-glutamyl transpeptidase, *TBIL* total bilirubin, *ALB* albumin, *PLT* platelet, *PT* prothrombin time, *UIBC* unsaturated iron binding capacity, *TIBC* Total iron binding capacity, *TSI* transferrin saturation index

### Correlations between iron-metabolism-related variables and the clinical characteristics of patients

The correlations between iron-metabolism-related variables and clinical characteristics are summarized in Table 3. As expected, serum iron levels were positively correlated with ferritin and TSI but negatively correlated with transferrin, UIBC, and TIBC (*P* < 0.05). Serum iron, ferritin and TSI were positively correlated with age, TBIL, and fibrosis stage (*P* < 0.05), while transferrin, UIBC, and TIBC were negatively correlated with age, TBIL, and fibrosis stage (*P* < 0.05). Serum iron, ferritin and TSI were positively correlated with ALT, AST, ALP, and GGT (*P* < 0.05), while UIBC was negatively correlated with ALT, AST, ALP, and GGT (*P* < 0.05). Serum iron levels and TSI were negatively correlated with ALB and the PLT count (*P* < 0.05), while transferrin, UIBC, and TIBC were positively correlated with ALB and the PLT count (*P* < 0.05). TSI was positively correlated with PT (*P* < 0.05), while TIBC was negatively correlated with PT (*P* < 0.05).

**Table 3:** Correlations between iron metabolism related variables and clinical characteristics of patients

Variables statistics	Iron (μmol/L)		Transferrin (g/L)		Ferritin (μg/L)		UIBC (μmol/L)		TIBC (μmol/L)		TSI (%)	
	r	*P*	r	*P*	r	*P*	r	*P*	r	*P*	r	*P*
Age (years)	0.296	0.003	− 0.416	< 0.001	0.349	< 0.001	− 0.394	< 0.001	− 0.375	< 0.001	0.376	< 0.001
IgG (g/L)	0.080	0.438	0.062	0.544	0.043	0.675	− 0.057	0.577	0.003	0.978	0.083	0.420
ALT (U/L)	0.348	< 0.001	− 0.031	0.765	0.418	< 0.001	− 0.311	0.002	− 0.091	0.375	0.332	0.001
AST (U/L)	0.462	< 0.001	− 0.097	0.346	0.460	< 0.001	− 0.415	< 0.001	− 0.107	0.299	0.453	< 0.001
ALP (U/L)	0.310	0.002	− 0.029	0.776	0.209	0.040	− 0.251	0.013	− 0.080	0.437	0.299	0.003
GGT (U/L)	0.408	< 0.001	− 0.104	0.312	0.340	0.001	− 0.350	< 0.001	− 0.136	0.186	0.390	< 0.001
TBIL (μmol/L)	0.498	< 0.001	− 0.220	0.030	0.395	< 0.001	− 0.526	< 0.001	− 0.249	0.014	0.533	< 0.001
ALB (g/L)	− 0.392	< 0.001	0.344	0.001	− 0.271	0.007	0.533	< 0.001	0.405	< 0.001	− 0.515	< 0.001
PLT (× 10^9^/L)	− 0.201	0.048	0.345	0.001	− 0.186	0.069	0.329	0.001	0.394	< 0.001	− 0.308	0.002
PT (s)	0.154	0.132	− 0.176	0.085	0.159	0.119	− 0.207	0.042	− 0.224	0.028	0.235	0.021
Iron (μmol/L)	NA	NA	− 0.439	< 0.001	0.583	< 0.001	− 0.859	< 0.001	− 0.244	0.016	0.933	< 0.001
Transferrin (g/L)	− 0.439	< 0.001	NA	NA	− 0.407	< 0.001	0.698	< 0.001	0.719	< 0.001	− 0.606	< 0.001
Ferritin (μg/L)	0.583	< 0.001	− 0.407	< 0.001	NA	NA	− 0.604	< 0.001	− 0.328	0.001	0.623	< 0.001
UIBC (μmol/L)	− 0.859	< 0.001	0.698	< 0.001	− 0.604	< 0.001	NA	NA	0.652	< 0.001	− 0.965	< 0.001
TIBC (μmol/L)	− 0.244	0.016	0.719	< 0.001	− 0.328	0.001	0.652	< 0.001	NA	NA	− 0.486	< 0.001
TSI (%)	0.933	< 0.001	− 0.606	< 0.001	0.623	< 0.001	− 0.965	< 0.001	− 0.486	< 0.001	NA	NA
Fibrosis stage	0.242	0.017	− 0.254	0.012	0.395	< 0.001	− 0.278	0.006	− 0.227	0.025	0.292	0.004

*NA* not applicable

*IgG* immunoglobulin G, *ALT* alanine aminotransferase, *AST* aspartate aminotransferase, *ALP* alkaline phosphatase, *GGT* gamma-glutamyl transpeptidase, *TBIL* total bilirubin, *ALB* albumin, *PLT* platelet, *PT* prothrombin time, *UIBC* unsaturated iron binding capacity, *TIBC* Total iron binding capacity, *TSI* transferrin saturation index

### Analysis of risk factors associated with advanced liver fibrosis

Binary logistic regression analysis was used to explore the risk factors associated with advanced liver fibrosis in AIH patients (Table 4). Patients with advanced liver fibrosis were older (*P* = 0.016); had higher serum IgG (*P* = 0.012), TBIL (*P* = 0.029), ferritin (*P* = 0.002), and TSI (*P* = 0.011); had longer PTs (*P* < 0.001) and lower PLT counts (*P* < 0.001); and had lower serum ALB (*P* = 0.039), transferrin (*P* = 0.043), UIBC (*P* = 0.008), and TIBC (*P* = 0.006) in the univariate logistic regression analysis. However, after the multivariate logistic regression analysis, the IgG, PLT count, PT, and ferritin were independent risk factors for predicting advanced liver fibrosis in AIH patients, with odds ratios (ORs) (95% confidence intervals [CIs]) of 1.123 (1.023, 1.232), 0.988 (0.979, 0.997), 1.758 (1.143, 2.704), and 1.002 (1.001, 1.004), respectively.

**Table 4:** Univariate and multivariate analysis of factors associated with advanced liver fibrosis

Variables	Univariate analysis		Multivariate analysis	
	OR (95% CI)	*P*	OR (95% CI)	*P*
Age (years)	1.053 (1.010, 1.097)	0.016		
Female	0.960 (0.312, 2.955)	0.943		
Autoantibodies positive	2.674 (0.694, 10.302)	0.153		
IgG (g/L)	1.084 (1.018, 1.154)	0.012	1.123 (1.023, 1.232)	0.014
ALT (U/L)	1.000 (0.999, 1.001)	0.820		
AST (U/L)	1.001 (0.999, 1.002)	0.314		
ALP (U/L)	1.006 (0.999, 1.014)	0.104		
GGT (U/L)	1.002 (0.999, 1.004)	0.273		
TBIL (μmol/L)	1.006 (1.001, 1.012)	0.029		
ALB (g/L)	0.921 (0.851, 0.996)	0.039		
PLT (× 10^9^/L)	0.984 (0.976, 0.992)	< 0.001	0.988 (0.979, 0.997)	0.013
PT (s)	1.876 (1.317, 2.673)	< 0.001	1.758 (1.143, 2.704)	0.010
Iron (μmol/L)	1.023 (0.994, 1.054)	0.125		
Transferrin (g/L)	0.494 (0.250, 0.977)	0.043		
Ferritin (μg/L)	1.002 (1.001, 1.003)	0.002	1.002 (1.001, 1.004)	0.012
UIBC (μmol/L)	0.969 (0.947, 0.992)	0.008		
TIBC (μmol/L)	0.943 (0.905, 0.984)	0.006		
TSI (%)	1.023 (1.005, 1.041)	0.011		

*OR* odds ratio, *CI* confidence interval, *IgG* immunoglobulin G, *ALT* alanine aminotransferase, *AST* aspartate aminotransferase, *ALP* alkaline phosphatase, *GGT* gamma-glutamyl transpeptidase, *TBIL* total bilirubin, *ALB* albumin, *PLT* platelet, *PT* prothrombin time, *UIBC* unsaturated iron binding capacity, *TIBC* total iron binding capacity, *TSI* transferrin saturation index

### Analysis of the diagnostic performance of ferritin using the ROC curve

**Fig. 1:**
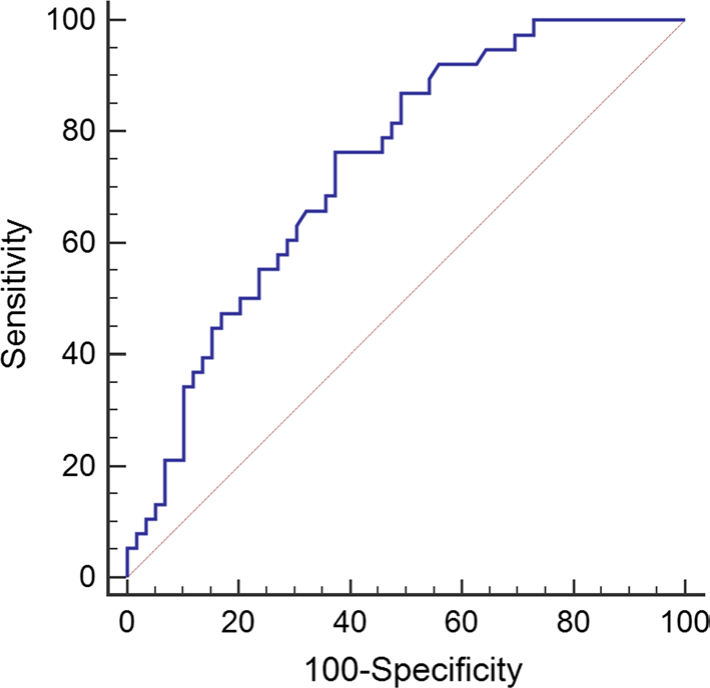
Ferritin ROC curve analysis by the DeLong method. ROC curve of ferritin for the prediction of advanced liver fibrosis in AIH. *ROC* receiver operating characteristic, *AIH* autoimmune hepatitis

The ROC curve showed that the best ferritin cut-off value was 199 μg/L, with 76.32% sensitivity and 62.71% specificity for the diagnosis of advanced liver fibrosis (area under the curve [AUC] = 0.738, 95% CI 0.639‐0.822; Fig. 1). Therefore, we compared the general characteristics of patients according to this cut-off level of ferritin. The age, ALT, AST, TBIL, iron, TSI, inflammatory grade, and fibrosis stage in the higher ferritin level group were significantly higher than those in the lower ferritin level group (P < 0.05), while transferrin, UIBC, and TIBC in the higher ferritin level group were significantly lower than those in the lower ferritin level group (P < 0.05). Significant differences between the two groups were not observed for other parameters (P > 0.05), as shown in Table 5.

**Table 5:** General characteristics of the patients by ferritin level

Variables	Ferritin ≤ 199 (μg/L) (n = 46)	Ferritin > 199 (μg/L) (n = 51)	*P*
Age (years)	49.6 ± 11.7	57.9 ± 9.4	< 0.001
Female, n (%)	41 (89.1)	41 (80.4)	0.235
Autoantibodies positive, n (%)	38 (82.6)	45 (88.2)	0.431
IgG (g/L)	16.50 (13.38, 21.80)	16.80 (12.90, 21.00)	0.694
ALT (U/L)	50.0 (23.0, 152.8)	124.0 (44.0, 482.0)	0.006
AST (U/L)	64.0 (28.0, 179.3)	209.0 (47.0, 542.0)	0.003
ALP (U/L)	101.0 (78.8, 148.0)	118.0 (72.0, 151.0)	0.549
GGT (U/L)	110.5 (63.5, 174.3)	147.0 (56.0, 301.0)	0.116
TBIL (μmol/L)	15.4 (11.7, 32.2)	25.4 (14.6, 133.7)	0.009
ALB (g/L)	41.1 ± 5.6	39.3 ± 5.5	0.106
PLT (× 10^9^/L)	195.4 ± 79.4	175.3 ± 60.5	0.162
PT (s)	12.9 ± 1.3	13.4 ± 1.6	0.09
Iron (μmol/L)	21.0 ± 12.1	33.7 ± 13.7	< 0.001
Transferrin (g/L)	3.04 (2.41, 3.40)	2.41 (2.13, 2.88)	0.001
UIBC (μmol/L)	45.0 (30.2, 58.6)	24.6 (12.5, 32.0)	< 0.001
TIBC (μmol/L)	64.9 (54.9, 75.4)	56.6 (53.6, 66.9)	0.022
TSI (%)	30.9 (19.1, 50.2)	56.9 (43.3, 80.8)	< 0.001
Histology (inflammation), n (%)			< 0.001
G1	2 (4.3)	1 (2.0)	
G2	30 (65.2)	12 (23.5)	
G3	12 (26.1)	28 (54.9)	
G4	2 (4.3)	10 (19.6)	
Histology (fibrosis), n (%)			0.001
F0	10 (21.7)	1 (2.0)	
F1	13 (28.3)	9 (17.6)	
F2	14 (30.4)	12 (23.5)	
F3	5 (10.9)	16 (31.4)	
F4	4 (8.7)	13 (25.5)	

Data are expressed as mean (± standard deviation), median (quartile 25, quartile 75) or number (proportion)

*IgG* immunoglobulin G, *ALT* alanine aminotransferase, *AST* aspartate aminotransferase, *ALP* alkaline phosphatase, *GGT* gamma-glutamyl transpeptidase, *TBIL* total bilirubin, *ALB* albumin, *PLT* platelet, *PT* prothrombin time, *UIBC* unsaturated iron binding capacity, *TIBC* total iron binding capacity, *TSI* transferrin saturation index

## Discussion

To the best of our knowledge, the association between iron-metabolism-related variables and liver fibrosis has not been extensively studied in AIH patients. In this study, we found that higher serum ferritin levels were independently associated with advanced liver fibrosis in patients with treatment-naive autoimmune hepatitis. AIH is an immune-mediated chronic, progressive inflammatory liver disease with diverse clinical manifestations. Liver fibrosis is a common complication in the progression of AIH, and many patients have advanced liver fibrosis at presentation [32]. Hence, the determination of liver fibrosis is also crucial for the determination of the prognosis of AIH and for the selection of treatment. These findings reveal the influence of ferritin on hepatic fibrosis in AIH patients. Serum ferritin is a noninvasive indicator that can be easily evaluated in clinical laboratories. Therefore, serum ferritin can be used as a routine reference index for monitoring hepatic fibrosis in all AIH patients.

Iron is derived from dietary sources containing haem (≤ 10%) and nonheme (> 90%) products [33, 34]. Extracellular ferrous iron is oxidized to ferric iron by oxidizing ferroxidase (hephaestin), and inactive ferric iron is bound to transferrin [35]. Transferrin is a protein synthesized and released by the liver and is responsible for iron transport through the plasma. In general, transferrin is increased in absolute iron deficiency to maximize the use of the limited available iron [36]. However, during inflammation, the ability of the liver to synthesize proteins, including transferrin, is reduced. UIBC and TIBC can be used as alternative tests for transferrin [37]. Similarly, we found that serum transferrin levels were positively correlated with UIBC and TIBC. As part of a complex system that senses plasma iron levels, transferrin can also regulate the activity of hepcidin [38]. Hepcidin is mostly produced by hepatocytes in response to iron load in cells. Ferritin is the main storage protein for iron, and the liver is the main ferritin storage site [39]. Moreover, ferritin is an acute phase protein that can be increased in the process of inflammation, whereas transferrin is downregulated in the process of inflammation and reduced in advanced liver disease [40–42], which is in accordance with our results. In addition, TSI is essential for identifying hepatic iron overload, and Ribot et al. reported that TSI was related to liver function damage and inflammation [43], which was partially consistent with our results.

Disturbances in iron regulation have been reported in diverse chronic liver diseases other than hereditary haemochromatosis [16–20]. Serum ferritin levels are increased in patients with chronic hepatitis B [44], chronic hepatitis C [45–47], chronic alcoholic liver disease [48], nonalcoholic fatty liver disease [25, 26, 48], and AIH [21, 22]. Disturbances in iron homeostasis in chronic liver diseases may be a manifestation of active inflammation and parenchymal damage [18, 40, 49] or a pathological mechanism by which iron toxicity aggravates liver injury and outcome [39, 50]. Iron overload can lead to oxidative stress, which has been implicated in the pathogenesis of many chronic liver diseases [51–54]. The production of reactive oxygen species can impair the cell membrane, damage mitochondrial function, affect gene expression, induce hepatocyte injury, and promote liver fibrosis [55].

Serum ferritin levels and serum iron concentrations were found to be increased in 65% and 58% of patients with untreated AIH [21]. Serum ferritin levels paralleled serum aminotransferase levels in patients with AIH and chronic hepatitis C [21, 56], which was consistent with our study’s findings. The iron disturbances in untreated AIH may reflect increased iron release from damaged hepatocytes, and their subsequent resolution with treatment‐induced normalization of aminotransferase and IgG reflects their remediation after successful treatment [21, 40]. The increase in liver iron promotes the synthesis of ferritin [57], which can activate the production of collagen and liver fibrogenesis [58]. More specifically, ferritin can activate hepatic stellate cells, which are key effectors of fibrogenesis. Although several prior studies have reported significant relationships between ferritin levels and liver fibrosis [24, 58–60], others have reported disparate findings [46, 60]. Therefore, we conducted this study to assess whether serum ferritin (and/or other iron-metabolism-related variables) was related to advanced liver fibrosis in AIH patients.

We also demonstrated that a higher serum IgG, lower PLT count, and longer PT were independent risk factors to predict advanced liver fibrosis in AIH patients. Our findings share many similarities with findings from several prior publications [4, 61–64]. Therefore, we believe that patients with higher serum ferritin, higher serum IgG, a lower PLT count, and a longer PT are more likely to have advanced liver fibrosis.

This study has a few limitations. First, the number of patients is relatively small because of the low prevalence of AIH. Sample size determination through calculations and statistical power determination are important in the validation of study findings. However, we did not formally determine our sample size through calculations, as our sample size was determined by the availability of existing data. Second, because this was a cross-sectional study, we could not demonstrate a causal link between ferritin and the risk of advanced liver fibrosis. In addition, the retrospective nature of the study raises the possibility of bias. Despite these limitations, our study investigated the relationship between ferritin levels and advanced liver fibrosis in AIH patients to determine whether serum ferritin could be used as a routine reference index for monitoring hepatic fibrosis in AIH patients. Further prospective large-scale studies are needed to confirm the causal relationship.

In summary, we showed that higher serum ferritin was independently associated with advanced liver fibrosis among patients with treatment-naive AIH, especially if combined with thrombocytopenia, a prolonged PT, and increased IgG. Serum ferritin may be used to predict the presence of advanced liver fibrosis in the clinic, but additional investigations are warranted for validation.

## Data Availability

The datasets used and/or analysed during the current study are available from the corresponding author upon reasonable request.

## Abbreviations

AIH: Autoimmune hepatitis
IgG: Immunoglobulin G
ALT: Alanine aminotransferase
AST: Aspartate aminotransferase
ALP: Alkaline phosphatase
GGT: Gamma-glutamyl transpeptidase
TBIL: Total bilirubin
ALB: Albumin
UIBC: Unsaturated iron binding capacity
TIBC: Total iron binding capacity
TSI: Transferrin saturation index
PT: Prothrombin time
PLT: Platelet
OR: Odds ratio
CI: Confidence interval
